# Report of the Select Committee of the House of Representatives, Relative to the Legalizing of the Study of Anatomy

**Published:** 1832-01-01

**Authors:** 


### VIII.

Report of the Select Committee of the House of Representatives, on so much of the Governor's Speech at the June Session, 1830, as relates to Legalizing the Study of Anatomy. Reported by a *Select Committee.* Octavo, pp. 118. Boston, 1831.

Of the paramount importance of anatomy to the profession, as well as to the public, we need now say nothing. To argue on the question would be preposterous at this period, when the discussions which occupied men's minds are still fresh and uneffaced, the arguments adduced on either side still ringing in our ears. The sensible and well-educated portion of our countrymen are sufficiently convinced of the necessity for anatomical knowledge; and if the vulgar of all ranks still allow ancient prejudices to blind their eyes, we may hope that they will be dispelled, at no distant epoch, by the advancing light of enquiry and intelligence. That this desirable event will take place, none who watch the signs of the times can doubt. The shadows of the darkness of the middle ages are fading around us, and the enemies of improvement and opponents of reform are beginning to merge and vanish in the gloom. Truth has prevailed and she will prevail; and if justice be upon our side, if reason sanctify our cause, we shall surely triumph, though the day of victory be delayed.

We are not amongst those who would scout and defy the honest prejudices of the people—prejudices nurtured by ages, and hallowed by their finer feelings. Least of all would we mock their prejudices on the subject of anatomy. If there be in human nature one trait of character which derives a more pure and holy origin than another, it is the reverence held by the living to the dead, and the tender regard which shrinks at an injury, merely apparent though it be, inflicted on the deceased. Far be it from us, and from our cause, to deride or to oppose this sacred sentiment, the source of much that is good and great, which binds the child to the parent, and makes the father live in his offspring; far be it from us to discountenance respect for the memory of the departed.

It should be, and indeed it is, the object of those who wish well to anatomy, to convince the public that the profession do pay regard to their feelings, and, because they do so, that they appeal in the manner they have done. The profession would save the living, by advancing the science they profess, and they would do it, as far as possible, without disrespect to the dead. It is the present shocking and degrading system, necessarily adopted for the acquisition of bodies for dissection, which insults society and lacerates its feelings. It is the present system which is calculated to carry horror, and loathing, and wretchedness into the bosom of families, which violates the grave, scathes every domestic affection, raises terror and panic in the public mind, and last, though not least, gives birth and nurture to a race of ruffians.

The subject has attracted or is attracting attention in most of the civilized countries of the world, and we venture to predict that at no remote period it will become an object of careful and anxious legislation in all. Medicine is a science of such essential consequence to the happiness of mankind, that all which concerns it must, sooner or later, be an object of solicitous research. Men see the importance of anatomy; even now they are busied in devising the means of rendering its attainment as little repugnant as may be. In America, the land of freedom, and therefore that in which the prejudices of the multitude can find, if excited, a loud and indisputable expression, the Legislative Assembly have made anatomy the theme of their deliberations and the subject of enactments. We have before us a report of a select committee of the House of Representatives which does credit to the information, the good sense and the liberality of those who drew it up. Some of our hereditary legislators might usefully brook a lesson from the popular delegates of America.

The report of the committee was given into the House on the 6th of January, 1831, and purports to be grafted on the Governor's speech at the June session, 1830. The committee propose to consider—

"I. The Rise and Progress of Anatomical Science:

II. Its indispensable importance to both great branches of the Healing Art; —the practice of Medicine and Surgery:

III. The interest which Society at large, especially, and the Medical Profession incidentally, have in the modification of the laws of this Commonwealth, so as to afford a reasonable facility for the pursuit of Anatomical Science:

IV. The Provisions, and the Character and Effect, of our present laws, regulating the practice of Physic and for the Protection of the Sepulchres of the Dead:

V. The Provisions that have been made in France and other enlightened countries, for the promotion of Anatomical Science; and

VI. Present those conclusions, which the Committee recommend for Legislative sanction by legal enactments, with a view to like results in our enlightened Commonwealth." 5.

They proceed to their task with industry and ability. The gradual rise of anatomical science is shewn with succinctness and precision, and the people of the United States, as of the old world also, may see in the details of its progress, how great discoveries have ever been the work of more than one age, at all events of many individuals. The earlier anatomists were not ripe for the discovery of the circulation of the blood; they could not make it. It was only when dissection had made progress, when Fabricius had demonstrated the valves of the veins, and *perhaps something else,* that Harvey, a student under Fabricius in his Paduan school, worked out the discovery a little farther, and disclosed to the world the circulation of the blood. Thus it is on all occasions that the most original thinkers and reasoners have had their pioneers. From a history of anatomy we also learn how its sober truths dispel the theories and dogmatism of the schools. Before anatomy was cultivated, men credulously received the fancies of Asclepiades, Galen, and Paracelsus; one futile system overthrew another; and the waste of human life and destruction of human comfort, by the earlier sects of physicians, must have been enormous.

Having traced the history of anatomical science, the committee next endeavour to prove that the study and knowledge of anatomy are essential to the safe and successful practice of medicine. The arguments which they employ are well calculated for producing a powerful impression on a mixed legislative assembly, but unnecessary to the readers of a medical journal. The following short quotation will shew the style in which the question is treated, and will perchance offer an useful hint to some of our own print-loving students.

"Would any one trust a valuable watch—an heir-loom—that in addition to its intrinsic value had a sort of moral and peculiar worth, from its having been the favorite timepiece of a beloved parent, of a departed sage or patriot—would such a valuable relic be trusted even to Roskell himself, had he no other knowledge than was to be learned from finely written descriptions, nicely executed and colored drawings, or even the best and most accurate models; to which however the principle of motion had never been imparted? Would a wise man employ to build the house, in which he meant to say, soul take thy rest, and enjoy the many good things that Providence has allotted thee, a house-builder whose knowledge of his trade had been learned from the works of Palladio or Stuart, rather than one, who, with cultivated mind, had learned the necessary details of his trade over the bench and in his workshop?

And shall we absurdly trust to operate on the human frame, made in God's own likeness, filled with that nice machinery, to which even to approximate would bid defiance to human ingenuity and industry, those individuals who are prepared by the study of only wax models and paper drawings? If we do, accident and good luck may sometimes lead the surgeon to happy results. But he has no certainty in his operation and feels no confidence in himself. He gropes in the dark. He may do this successfully so far as to pass through the great avenues without losing himself.

But how can we expect him with accuracy and celerity to pick up a cambric needle or to remove a grain of sand, not merely from the surface, but from the remotest and obscurest corner of that surface?

It would be far safer and wiser for a ship's crew to trust themselves and their vessel to a blind pilot; for if they took a true departure and knew the course and distance to the place at which they meant to arrive, their chance for a fortunate issue would be far greater." 27.

The Committee, however, can give some information of consequence to medical men themselves. Thus we find them incidentally allude to "a secret worth knowing," a cure for dyspepsia.

"Within the last twenty-five years we have heard very much of Dyspepsia, a malady of general prevalence and of afflictive results. Quite recently, a cure is said to have been discovered for it by Mr. O. Halstead, a bookseller of New-York city, who has published an interesting account of his process. An examination of this publication has satisfied the committee, that without the lights thrown by anatomical science on the human economy, Mr. Halstead's mode of cure would not have been discovered. His mode consists:

"1st. In a relaxation of the external muscles of the abdomen by external applications; and 2dly, in applying external excitement through the nervous system to the coats of the stomach. Had he not been generally acquainted with the anatomical structure of man, he would never have been brought to the conclusions, he is said so successfully to have applied to practice; and if his theory be sound, he has been most fortunate in possessing enough sagacity to draw the true inferences from the premises, anatomy had supplied him." 43.

We are afraid that Mr. Halstead's cures are not likely to confer great glory on the cause in the service of which they are enlisted. But this by the way. The Committee justly dwell on the important and invincible truth, that the judicious legalization of anatomy is calculated to prevent the feelings of friends from being harrowed, by avoiding the indecent exposures necessarily entailed on society by the system of exhumation. The feelings of relatives and friends are not to be contemned but respected ; the associations which encircle the memory of the dead are to be consecrated, not disturbed. The Committee next inquire, who has the greatest interest in facilitating the study of anatomy, the profession or the public? The latter most decidedly. Be physicians and surgeons anatomists or not, disease and accident will come, and men, when afflicted with corporeal ills, will fly for assistance to art, however imperfect it may be. It is true that, without anatomy the profession would be less scientific; its members would not practise according to knowledge, but kill according to rule; yet still they must be employed, and still they must be paid. It is idle and preposterous to talk of medicine alone being the gainer by anatomical pursuits. In Massachusetts there is no direct law upon the subject of anatomical dissections.

"But there are two laws, which have an important, though not an immediate bearing on this subject. In 1815 a law was passed for the protection of the sepulchres of the dead, which punished the exhumation of any dead body, or the knowingly and wilfully receiving, concealing or disposing of any such dead body, by a fine of not more than S. 1000, or imprisonment for not more than one year.

Before the passing of this act, several cases at common law were brought before the Supreme Judicial Court ; in all of which, where there was a conviction, the party was punished. Where it appeared that the exhumation was for subjects for Dissection, a small fine was imposed. The last case of this kind was against a now eminent physician, then of Essex County, in which several important law points were raised, but the case does not appear to have been reported. Under the statute, there have been several prosecutions, convictions and punishments. A similar statute exists in New-Hampshire and in Vermont. In New-Hampshire the punishment is by fine, not to exceed S. 2000, whipping not to exceed 39 stripes, or imprisonment not to exceed one year—all or any of these punishments to be inflicted at the discretion of the Court.

The Vermont statute varies from that of New-Hampshire only in the fine, which is S. 1000 instead of S. 2000. The other provisions are the same." 63.

Thus the law is severe against exhumation, the only possible means of obtaining subjects for dissection; and yet, with the same tyrannical inconsistency as that displayed in our own code, another law has been enacted, compelling every practitioner of medicine to obtain a degree or a licence before he may venture to practise—that degree or licence not being obtainable without the display of minute anatomical knowledge. The price of subjects in Boston is double what it was ten years ago, and from fifteen to twenty subjects are annnally required for the medical school—the price being from S. 15. to S. 20. each.

"The only legalized mode of supplying subjects for dissection is the sentence or order of the Supreme Judicial Court of this state, and of the Circuit Court of the United States, in capital convictions within their respective jurisdictions. The insufficiency of this supply may be inferred from the statements of the secretary of the Commonwealth, and of the clerk of the United States District Court. The secretary of the Commonwealth states, in answer to inquiries addressed him by the chairman of this committee, that the whole number of executions or suicides of convicts from January 1, 1800, to December 31, 1830, is but 26, less than one a year. The clerk of the United States District Court, in reply to like inquiries from the chairman states, that from the adoption of the Federal constitution and the first organization of the Federal Courts, down to the present time, the whole number of executions and of suicides of convicts, sentenced by that Court in this District, is but fourteen, being about one in three years. The absolute inadequacy of this supply, from either or both of these sources for any useful purpose, added to the consideration that the infliction of dissection as a part of the sentence in capital cases, has done much to create, and will do much to continue, the prejudice against anatomy existing in the community, has suggested to your committee the expediency of a different provision from that now existing, in relation to the dead bodies of those capitally convicted, so far as the subject is within the control of the State legislation. If dissection be no longer inflicted by our State Courts, as a terror and disgrace, —full and free discussion of this interesting and important topic may, we hope, soon disabuse the public mind of its worst prejudices in relation to it. Your committee cannot doubt, that the deservedly eminent judge and scholar, who holds the United States courts in this Judicial District, will so far as his important influence extends on this and every other subject, be found against unfounded prejudice, and on the side of liberal science and true philosophy." 68.

The Committee take a survey of the state of the laws on anatomical investigations in England and France. In the latter country, they challenge the approbation of all well-informed persons.

" It now becomes the duty of your Committee to present their own conclusions, on this whole matter, which they would recommend to the sanction of the General Court. They may be thus stated : That—

Ist. Anatomy is an important science, whose successful cultivation and improvement is of essential interest to all classes of the population of this Commonwealth.

IId. Dissection for anatomical purposes is highly laudable, and deserving of public encouragement, so far as it can be done without violence to the feelings of surviving relatives or friends.

IIId. That the Laws of the Commonwealth, which now act indirectly on the study of anatomy, require change, and that the study of anatomy should be legalized.

I. For this purpose the Committee propose so far to alter the statute of 1815, for the 'protection of the sepulchres of the dead' as to authorize the proper Municipal authorities, in the city of Boston, and in the several towns of the Commonwealth, to deliver to any physician, regularly licensed according to the laws of this Commonwealth, such dead bodies as may be required to be buried at the public expense and which shall not be claimed by any *one* person, *whether kin, or friend, or acquaintance,* within twenty-four hours from and after death. This permission should be accompanied with restrictions, that the physician so receiving a subject, after he had used it for scientific research, should be bound to have its remains properly interred, with the religious funeral rites, that a Christian people ought to require and must approve.

II. The proviso, authorising the Courts to dispose of bodies of executed criminals for dissection should be repealed.

III. That the penalty for disinterring dead bodies or for receiving them, knowing them to have been so disinterred, should be increased, so as effectually to guard against any attempt to transcend those limits for the supply of anatomical subjects, which this enlightened Legislature may designate. These views the Committee have combined in a bill, which is appended to this Report." 74.

It has been urged in America, as it has been urged in this country, that by consigning the bodies of the unclaimed poor to the purposes of dissection, poverty is branded as a crime. Undoubtedly the argument is specious, and the case seems hard. But much is to be urged in defence of the measure. If dissection be absolutely and indispensably required, which it can be and has been proved to be, it necessarily follows, that the bodies of some class of persons must be taken for the purpose. By repealing the law which makes dissection part and parcel of the punishment for crime, it will lose its disgraceful character, and become merely an imaginary hardship. But still it will be a hardship, and to whom? to the dead? it matters not to them. By taking the bodies of unclaimed paupers only, the feelings of friends are not wounded, and the only individual who can feel a pang is the dying pauper himself. Those who see much of death in the lowest classes will probably admit that, with such a person, such consideration will commonly have little weight. If they think of it at all, they will think of it as an evil inseparable from their lot, and not to be ranked with the many positive ills they suffer. It must not be forgotten that the unclaimed poor, in our hospitals and alms-houses, are mostly those whose lives have been vicious and characters abandoned—the prostitute, the vagrant, the sot. If any are to suffer after death for the community, it should surely be those who have contributed evil rather than good to it when living. At last, indeed, the dispute resolves itself to this :—Dissection being necessary, the legislature must provide it, at the lowest possible expense of individual hardship and general disgust. As far as the wit of man has hitherto been able to discover, the appropriation of the unclaimed poor, when dead, to its purposes, is the best and most reasonable way of meeting the difficulty.

" It appears from a statement obtained at the Boston Health Office, that from January 1, 1830, to December 21, inclusive, there were 194 burials at the expense of the City, and also in addition, 88 burials in the Tombs of the House of Industry at South Boston. The number of burials of the same description in other towns of the Commonwealth would be considerable, but less in proportion to the population. There can be no doubt the supply of subjects would be ample." 77.

The Committee, having terminated their Report, recommended to the legislature of Massachusetts a bill, which, with some modifications, the Legislature adopted. It passed the House, received the Governor's signature, and is now a law. The profession and the public on this side the Atlantic may see, by the Act in question, that America has, on this occasion, led the way in the march of liberality and improvement.

" SENATE, No. 21.

The Amendments of the House of Representatives are printed in Italics, and were all moved by the Chairman, J. B. Davis, Esq. to meet the views of the House.

### Commonwealth of Massachusetts.

In the Year of our Lord One Thousand Eight Hundred and Thirty One.

### AN ACT

More effectually to Protect the Sepulchres of the Dead, and to legalize the Study of Anatomy in certain cases.

SECT. 1. *Be it enacted by the Senate and House of Representatives in General Court assembled, and by the authority of the same,* That if any person, not being authorized by the Board of Health, Overseers of the Poor, or Selectmen in any town of this Commonwealth, or by the Directors of the House of Industry, Overseers of the Poor or Mayor and Aldermen of the City of Boston, in said Commonwealth, shall knowingly or wilfully dig up, remove or convey away, or aid and assist in digging up, removing, or conveying away, any Human body, or the remains thereof, such person or persons so offending, on conviction of such offence in the Supreme Judicial Court of this Commonwealth, shall be adjudged guilty of felony, and shall be punished by solitary imprisonment for a term not exceeding ten days, and by confinement afterwards to hard labour for a term not exceeding one year; or shall be punished by a fine not exceeding two thousand dollars to enure to the benefit of the Commonwealth, and by imprisonment in the common jail for a term not exceeding two years at the discretion of the Court, according to the nature and aggravation of the offence.

Sect. 2. *Be it further enacted,* That if any person shall be in any way, either before or after the fact, accessary to the commission, by any person or persons of the offence described in the first section of this act, such person or persons shall be adjudged and taken to be principals, and shall be on conviction in the Court aforesaid, subject to the same punishments and forfeitures as are in said first section provided.

Sect. 3. *Be it further enacted,* That from and after the passing of this Act, it shall be lawful for the Board of Health, Overseers of the Poor, and Selectmen of any town in this Commonwealth and for the Directors of the House of Industry, Overseers of the Poor, and Mayor and Aldermen of the City of Boston in said Commonwealth to surrender the dead bodies of such persons, *except Town Paupers,** as may be required to be buried at the public expense to any regular physician, duly licensed according to the Laws of this Commonwealth, to be by said physician used for the advancement of anatomical science; preference being always given to the Medical schools that now are or hereafter may be by law established in this Commonwealth, during such portions of the year as *such* schools or either of them may require subjects for the instruction of Medical Students: *Provided always,* That no such dead body shall in any case be so surrendered, if within *thirty-six* hours from the time of its death any one or more persons claiming to be kin, friend, or acquaintance to the deceased, shall require to have said dead body inhumed: *or, if it be made to appear to the Selectmen or Overseers of the Poor of any town in this Commonwealth, or to the Mayor and Aldermen or Overseers of the Poor of the City of Boston, that such dead body is the remains of a stranger or traveller, who suddenly died before making known, who or whence he was;* but said dead body shall be inhumed, and when so inhumed, any person disinterring the same *for purposes of dissection,* or being accessary as is described in the second section of this Act to *such* exhumation, shall be liable to the punishments and forfeitures in this Act respectively provided: *And provided further,* That every physician so receiving any such dead body, before it be lawful to deliver him, the same shall in each case give to the Mayor and Aldermen of the City of Boston, or to the Selectmen of any town of this Commonwealth, as each case may require, good and sufficient bond or bonds, that each body by him so received, shall be used only for the promotion of anatomical science; that it shall be used for such purposes only in this Commonwealth, and so as in no event to outrage the public feeling; and that after having been so used the remains thereof shall be decently inhumed.

Sect. 4. *Be it further enacted,* That from and after the passing of this Act, it shall be lawful for any physician duly licensed according to the Laws of this Commonwealth, or for any Medical Student under the authority of any such physician to have in his possession, to use and employ human dead bodies, or the parts thereof for purposes of anatomical inquiry or instruction.

---

* "Those who receive charity from the overseers of the poor, and die out of the alms-house, or House of Industry, as we term it."

Sect. 5. *Be it further enacted, That nothing in this Act, shall be so construed as to give to the Board of Health, Overseers of the Poor, or Selectmen of any town in this Commonwealth, or to the Directors of the House of Industry, Overseers of the Poor, or Mayor and Aldermen of the City of Boston, in said Commonwealth, any power to license the digging up of any dead human body, or the remains thereof, other than was possessed by them before the passing of this Act, or is given them by the third section of this Act.*

Sect. 6. *Be it further enacted,* That the Act passed March 2, 1815, entitled 'An Act to protect the Sepulchres of the Dead,' and also all other Acts or parts of Acts, contravening the provisions of this Act, be and the same hereby are repealed.

House of Representatives, Feb. 11, 1831.
Read three times and passed to be engrossed,
Sent up for concurrence,
P. W. WARREN, Clerk." 4.

It will be obvious to our readers, that the foregoing Act embodies in its provisions most of what has been desired in this country. So far as we can see, it leaves untouched the penal statute, which makes dissection part of the punishment for murder and other great crimes. Probably, this may be the object of a separate enactment. The Act of the Massachusetts Assembly appears to be straightforward and simple, and calculated to answer the ends proposed. It has infinitely the advantage over the legislative bantling which, we had almost said fortunately, miscarried in this country. The American law leaves no field for trickery; does not open a job here and erect corners for abuse there; does not constitute boards, and licenses, and committees; in fact it is not stained by that leaven of the patronage system, which seems to infect every action of every institution in this happy land. The American law simply provides bodies for dissection, and gives them first to the medical schools—to regularly-educated medical men when the schools have been supplied.

In conclusion, we beg to press one consideration on our medical brethren in town and country. At the present moment, foreigners are excelling us in the cultivation of our science, and there can be little question that this is owing to their superior facilities for the acquisition of anatomical knowledge. In America, where obstructions were thrown, as here, in the way of the profession, those obstructions are vanishing before the energetic appeals of that profession to a wise legislature. Let us imitate their example, and we shall equal their success. Let us vindicate for ourselves the claim of being heard, and let us shew that, by our perseverance, our consistency, and still more by the justice of our demands, we *will* be attended to. The victory is sure if we combine. If our Commons' House should be altered, and amended in its constitution, as most probably it will be, the voice of the middle classes will acquire a louder tone, and the profession can make its demands more audible. Petitions should be presented, the country and the London surgeon should enlist on their side the individual members of the legislature with whom they become professionally acquainted; and all should endeavour to dispel the prejudices which still reign in the minds of a portion of the public, a portion which we believe to be comparatively small, at least in the educated classes. Again we exhort the profession to bestir itself, to shake off its lethargy, to urge its wishes and desires at all proper opportunities: we exhort it to success. Liberal institutions are rising around us; the lion of freedom has roused himself, and its enemies are looking aghast. It is with science as it is with the political institutions of nations; she is withered and cramped by the trammels of oligarchies and of despotisms. Let her be free, say we, as the air we breathe, for the more she is released from her bondage, the more bold will be her gait, the more bright will be her hue.

